# Protective Effects of Hydrogen Gas on Experimental Acute Pancreatitis

**DOI:** 10.1371/journal.pone.0154483

**Published:** 2016-04-26

**Authors:** Hao-xin Zhou, Bing Han, Li-Min Hou, Ting-Ting An, Guang Jia, Zhuo-Xin Cheng, Yong Ma, Yi-Nan Zhou, Rui Kong, Shuang-Jia Wang, Yong-Wei Wang, Xue-Jun Sun, Shang-Ha Pan, Bei Sun

**Affiliations:** 1 Department of Emergency Surgery, The First Affiliated Hospital of Harbin Medical University, Harbin, People’s Republic of China; 2 Department of Pancreatic and Biliary Surgery, The First Affiliated Hospital of Harbin Medical University, Harbin, People’s Republic of China; 3 Department of Abdominal Ultrasound, The First Affiliated Hospital of Harbin Medical University, Harbin, People’s Republic of China; 4 Department of Diving Medicine, Second Military Medical University, Shanghai, People’s Republic of China; University of Szeged, HUNGARY

## Abstract

Acute pancreatitis (AP) is an inflammatory disease mediated by damage to acinar cells and pancreatic inflammation. In patients with AP, subsequent systemic inflammatory responses and multiple organs dysfunction commonly occur. Interactions between cytokines and oxidative stress greatly contribute to the amplification of uncontrolled inflammatory responses. Molecular hydrogen (H_2_) is a potent free radical scavenger that not only ameliorates oxidative stress but also lowers cytokine levels. The aim of the present study was to investigate the protective effects of H_2_ gas on AP both *in vitro* and *in vivo*. For the *in vitro* assessment, AR42J cells were treated with cerulein and then incubated in H_2_-rich or normal medium for 24 h, and for the *in vivo* experiment, AP was induced through a retrograde infusion of 5% sodium taurocholate into the pancreatobiliary duct (0.1 mL/100 g body weight). Wistar rats were treated with inhaled air or 2% H_2_ gas and sacrificed 12 h following the induction of pancreatitis. Specimens were collected and processed to measure the amylase and lipase activity levels; the myeloperoxidase activity and production levels; the cytokine mRNA expression levels; the 8-hydroxydeoxyguanosine, malondialdehyde, and glutathione levels; and the cell survival rate. Histological examinations and immunohistochemical analyses were then conducted. The results revealed significant reductions in inflammation and oxidative stress both *in vitro* and *in vivo*. Furthermore, the beneficial effects of H_2_ gas were associated with reductions in AR42J cell and pancreatic tissue damage. In conclusion, our results suggest that H_2_ gas is capable of ameliorating damage to the pancreas and AR42J cells and that H_2_ exerts protective effects both *in vitro* and *in vivo* on subjects with AP. Thus, the results obtained indicate that this gas may represent a novel therapy agent in the management of AP.

## Introduction

Acute pancreatitis (AP) is a serious disease that is associated with the release of digestive enzymes into the pancreatic interstitium and systemic circulation and that has an overall mortality rate of 10–25% [1]. Systemic inflammatory response syndrome (SIRS) is the most important contributor to multiple organ dysfunction syndrome (MODS) and death in the early stage of severe acute pancreatitis (SAP). The relationship between pancreatic injury and this uncontrolled systemic response is not completely understood. However, accumulating evidence indicates that the synergy between cytokines and oxidative stress is critically involved in the development of local and systemic complications associated with AP [2].

The serum levels of cytokines, such as TNF-α, IL-1β and IL-6, increase during the course of AP and appear to be correlated with the severity of pancreatic inflammation [3]. TNF-α is regarded as an initiator of the cytokine cascade because it induces the synthesis and release of other cytokines and participates in the activation of alveolar macrophages. Both pretreatment with an antibody against TNF-α and the blockade of TNF-α production with the drug pentoxifylline have been shown to ameliorate experimental AP [4]. Additionally, in knockout mice with TNF-α receptor deficiency, the rate of mortality due to AP is decreased because the systemic response is restrained [5].

The role of oxidative stress in AP has been demonstrated indirectly by the beneficial effects of antioxidants and directly by pancreatic glutathione (GSH) depletion and increased lipid peroxidation (LPO) [6]. Furthermore, circulating xanthine oxidase released by the damaged pancreas acts as a source of systemic oxidative stress that contributes to lung inflammation through a mechanism mediated by the up-regulation of the protein P selectin [7,8].

Cytokines and oxidative stress act synergistically in a vicious circle in the early stage of AP. Cytokines induce oxidative stress through different mechanisms and in turn, oxidative stress up-regulates cytokine gene expression [9]. Consequently, the interaction between cytokines and oxidative stress greatly contributes to the amplification of the uncontrolled inflammatory cascades in SIRS and MODS.

A series of recent studies showed that molecular hydrogen (H_2_) is a potent free radical scavenger that diffuses rapidly across cell membranes and can selectively reduce toxic free radicals, such as hydroxyl radicals (·OH) and peroxynitrite (ONOO^-^) [10]. H_2_ not only ameliorates oxidative stress but also lowers cytokine levels (TNF-α, IL-1β and IL-6) in several oxidative stress-induced diseases [11]. The properties of H_2_ indicate that it may confer protection against damage in AP. Therefore, this study was designed to elucidate the potential therapeutic effects of H_2_ on the interaction between cytokines and oxidative stress in the early stage of AP, both *in vivo* and *in vitro*.

## Materials and Methods

### Reagents

Monoclonal antibodies against TNF-α, IL-1β, IL-6, and IL-10 were purchased from Santa Cruz Biotechnology, Inc. (Santa Cruz, CA, USA). TNF-α, IL-1β, IL-6, and IL-10 solid-phase enzyme-linked immunosorbent assay (ELISA) kits were obtained from R&D Systems, Inc. (Minneapolis, MN, USA). RNA Extraction Kits and SYBR^®^ PrimeScript^TM^ RT-PCR Kits were purchased from Takara (Tokyo, Japan). Annexin V-FITC and propidium iodide (PI) were purchased from Biosea Biotechnology (China).

### AR42J cells

A murine pancreatic acinar cancer cell line (AR42J) was purchased from the American Type Culture Collection (Rockville, MD, USA), and the cells were cultured in Dulbecco’s modified Eagle’s medium (GIBCO BRL, Life Technologies, Inc., Grand Island, NY, USA) supplemented with 10% fetal bovine serum, glutamine (2 mM), penicillin (100 U/mL), and streptomycin (100 U/mL) in a humidified atmosphere of 5% CO_2_ at 37°C [12].

### Animals and experimental model of pancreatitis

All of the experiments were performed in accordance with the experimental protocol approved by the Committee for Research and Animal Ethics of Harbin Medical University, Harbin, China and complied with the criteria detailed in the US National Institutes of Health Guidelines for the Care and Use of Laboratory Animals.

Forty healthy male Wistar rats (250–300 g) were purchased from the Animal Center of the First Clinical Hospital of Harbin Medical University (Harbin, China). The animals were fed a commercial pellet chow and housed in cages under standard conditions at room temperature with a 12-h light–/12-h dark cycle. Prior to the start of the experiments, the animals were deprived of food but provided drinking water *ad libitum*.

The animals were anesthetized through an intraperitoneal injection of pentobarbital sodium (40 mg/kg body weight), and AP was induced as previously described [13]. Briefly, a midline laparotomy was performed under anesthesia, and a blunt 27-gauge needle was introduced into the distal end of the biliopancreatic duct via puncture of the duodenal wall. The proximal bile duct was temporarily occluded at the porta hepatis by a vascular clamp. Sodium taurocholate (5%; 20 mg/kg; Sigma-Aldrich, USA) was infused at a pressure of 5 kPa under the control of a sphygmomanometer. The duodenal and abdominal wounds were closed, and the animals were allowed to recover on heated pads. The control animals were subjected to a sham operation, which included laparotomy and separation of the pancreatobiliary duct.

### Experimental protocol

In the first set of experiments, AR42J cells were plated at a density of 2×10^6^/mL on a 100-mm culture plate (Falcon 3047; Becton Dickinson, Lincoln Park, NJ, USA) and allowed to attach for 12 h. The cells were then randomly divided into the following four groups: a C group, a Ce group, a C + H_2_ group and a Ce + H_2_ group. The cells in the C group were routinely cultured at 37°C in Dulbecco’s modified Eagle’s medium (GIBCO BRL, Life Technologies, Inc., Grand Island, NY, USA) supplemented with 10% fetal bovine serum, glutamine (2 mM), penicillin (100 U/mL) and streptomycin (100 U/mL) in an incubator with 95% air and 5% CO_2_. The cells in the Ce group were treated with cerulein at a concentration of 10^−8^ M [14,15] and incubated under the same conditions. The cells in the C + H_2_ group were maintained in a H_2_-rich medium, which was prepared as described previously, and the cells in the Ce + H_2_ group were treated with cerulein at a concentration of 10^−8^ M in a H_2_-rich medium [10]. Briefly, H_2_ gas was dissolved beyond saturation levels in the medium under a pressure of 0.4 MPa for 2 h. A high concentration of dissolved H_2_ (1.3±0.1 mg/L) was confirmed by gas chromatography (Biogas Analyzer BAS-1000, Mitleben, Osaka, Japan). The pH of the culture medium without H_2_ gas was 7.38±0.01, and that of the culture medium with H_2_ gas was 7.41±0.01. The cells of the last two groups were then incubated under the same conditions as those of the first two groups in an airtight incubator (Precision Scientific, Winchester, VA, USA) with 2% H_2_, 5% CO_2_, and balanced air. Samples of the four groups of cells and media were collected after 24 h of incubation. All of the tests were performed six times.

In the second set of experiments, the animals were randomly allocated into four experimental groups (n = 10 for each group): a Sham group, an AP group, a Sham + H_2_ group, and an AP + H_2_ group. The animals in the Sham group were administered anesthetic gas [sevofrane (1.5%) in N_2_O/O_2_ (70:30) gas] via inhalation using a TF-1 gas flowmeter (YUTAKA Engineering Corp., Tokyo, Japan) after sham operation. The AP group received the same treatment after the induction of AP. The rats in the Sham + H_2_ group were administered 2% H_2_ gas in the anesthetic gas supplied through the gas flowmeter (flow rate of 1 L/min) after sham induction, whereas the animals in the AP + H_2_ group received 2% H_2_ gas (flow rate of 1 L/min) after AP induction. Cylinders containing 2% H_2_ and balanced anesthetic gas were purchased from Harbin Liming Gas Co., Ltd. (Harbin, China). The treatment concentration of H_2_ was determined based on previous studies [10]. All of the animals were sacrificed 12 h after the induction of AP. After laparotomy and pancreatic samples were collected, 4 to 5 mL of arterial blood was obtained from the abdominal aorta. The blood samples were centrifuged at 3,000 rpm for 5 min to collect sera, which were stored at -20°C until assayed. Some pancreatic samples were processed immediately for real-time PCR analysis, whereas the others were either rinsed in saline buffer, snap-frozen in liquid nitrogen and stored at -80°C or fixed in 4% v/v neutral phosphate-buffered formalin and then embedded in paraffin wax for subsequent measurement.

### Cell survival assay

The AR42J cell viability was analyzed using the Cell Counting Kit-8 assay. The cells were seeded into 96-well plates and incubated overnight in a humidified CO_2_ incubator (95% air and 5% CO_2_) to allow the cells to adhere and recover. The culture medium was then replaced with either H_2_-rich or normal medium containing cerulein at a concentration of 10^−8^ M for 24 h as described above. The cells were then incubated with a WST-8 solution at 37°C for 1 h, and the absorbance at 450 nm was measured with a microplate reader (MPR-A4i, Tosoh Corporation, Tokyo, Japan). Cells cultured in normal medium served as controls. The cell viability index was calculated according to the following formula: experimental OD value/control OD value×100% [16]. The cells were viewed under a laser-scanning confocal microscope (LSM-510, Carl Zeiss Jena GmbH, Jena, Germany) to visualize those that had been injured and undergone necrosis. Annexin V was excited at 488 nm and emitted at 530 nm, and PI was excited at 536 nm and emitted at 617 nm. Five randomly selected microscopic fields were examined to determine the percentage of positive cells. All of the experiments were performed in triplicate and repeated three times.

### Determination of cytokine mRNA expression

Real-time quantitative RT-PCR was performed to assess the mRNA expression levels of TNF-α, IL-1β, IL-6, and IL-10 in AR42J cells. The total RNA from the cells was extracted using an RNA Extract Kit and converted to first-strand cDNA according to the manufacturer’s instructions. Quantitative real-time PCR was performed with an SYBR PrimeScript^TM^ RT-PCR Kit and a LightCycler System (Roche Diagnostics, Lewes, UK). The primer sequences used for PCR were as follows: TNF-α sense 5’-CCAGGAGAAAGTCAGCCTCCT-3’ and antisense 5’-TCATACCAGGGCTTGAGCTCA-3’, resulting in an 87-bp product; IL-1β sense 5’-CACCTCTCAAGCAGAGCACAG-3’ and antisense 5’-GGGTTCCATGGTGAAGTCAAC-3’, resulting in a 79-bp product; IL-6 sense 5’-CTGGTCTTCTGGAGTTCCGTTTC-3’ and antisense 5’-CATAGCACACTAGGTTTGCCGAG-3’, resulting in a 301-bp product; IL-10 sense 5’-GGCTCAGCACTGCTATGTTGCC-3’ and antisense 5’-AGCATGTGGGTCTGGCTGACTG-3’, resulting in a 116-bp product; and β-actin sense 5’-GAACACGGCATTGTAACCAACTGG-3’ and antisense 5’-GGCCACACGCAGCTCATTGTA-3’, resulting in a 77-bp product. Amplification was performed under the following conditions: 95°C for 30 s followed by 40 cycles of denaturation at 95°C for 5 s and annealing at 60°C for 20 s [17]. All reactions were performed in triplicate.

### Measurement of 8-hydroxydeoxyguanosine (8-OHdG) in the medium

8-OHdG is an indicator of oxidative DNA damage and a ubiquitous marker of oxidative stress. The 8-OHdG level in the medium was measured using an 8-Hydroxy-2-deoxyguanosine Enzyme Immunoassay Kit (Item No. 589320; Cayman Chemical Company, Ann Arbor, MI, USA). The spectrophotometer (DU 640B; Beckman Coulter, Inc., Fullerton, CA, USA) reading was obtained at 405 nm [18].

### Measurement of LPO

The malondialdehyde (MDA) level is widely used as an indicator of free radical-mediated LPO. Pancreatic tissue was homogenized in Tris-HCl (20 mmol; pH 7.4), and the tissue homogenate (500 μL) was then centrifuged at 2500 g and 4°C for 10 min. The supernatant (200 μL) or medium (200 μL) was then used to measure the MDA level (Lipid Peroxidase Assay Kit; Merck Biosciences GmbH, Darmstadt, Germany). The spectrophotometer reading was obtained at 586 nm [19].

### Cytokine assay

The levels of TNF-α, IL-1β, IL-6, and IL-10 in the medium and serum were assayed using commercial ELISA kits according to the manufacturers’ protocols.

### Biochemistry analysis

The medium amylase activity and the serum amylase and lipase activities, which are indicators of pancreatic dysfunction, were measured using an auto analyzer (Vitros 750 autoanalyzer, Johnson & Johnson, Rochester, NY, USA).

### Histological examination

The pancreatic tissue samples used for histological examination were immersed in 4% formaldehyde and embedded in paraffin. Hematoxylin-and-eosin-stained pancreatic sections were scored by two pathologists who were blind to the sample identities. Each section was scored based on the severity of pancreatitis on a scale of 0 to 4 (normal to severe) as previously described [17,20].

### Western blot analysis

The methodology used for Western blot analysis has been described previously [21]. Briefly, pancreatic tissues and cells were sonicated in RIPA buffer and homogenized. Debris was removed by centrifugation at 12,000 g and 4°C for 10 min. Samples containing 50 μg of protein were electrophoresed in polyacrylamide SDS gels and transferred to polyvinylidene difluoride (PVDF) membranes. The membranes were blocked with 3% BSA, incubated with primary antibodies and then with an alkaline phosphatase-conjugated secondary antibody and developed with 5-bromo-4-chloro-3-indolyl phosphate/nitro blue tetrazolium (Tiangen Biotech Co. Ltd., Beijing, China). The blots were also stained with an anti-β-actin antibody as an internal control for the amount of target proteins.

### Immunohistochemical analysis

Pancreatic sections were rinsed with phosphate-buffered saline, blocked with 3% bovine serum albumin (BSA) for 2 h, and incubated overnight with antibodies against TNF-α, IL-1β, IL-6, and IL-10. The sections were subsequently incubated for 30 min with a secondary antibody using an Ultra-Sensitive TMS-P Kit (Zhongshan Co., Beijing, China), and the immunoreactivity was visualized with SigmaFast 3,3’-diaminobenzidine tetrahydrochloride and CoCl_2_ enhancer tablets (Sigma-Aldrich). The sections were counterstained with hematoxylin, mounted, and examined by light microscopy. Positive cells were identified by the presence of a dark reddish-brown chromogen. Twenty randomly selected microscopic fields were examined blindly by a pathologist to determine the percentage of positive cells.

### Measurement of myeloperoxidase (MPO) activity in pancreatic tissues

The pancreatic MPO activity was determined using a chemical-based commercially available kit according manufacturer's instructions (Jiancheng Biochemistry Co., Nanjing, China). Pancreatic tissue (100 mg, wet weight) was homogenized in 2 mL of 10 mM phosphate buffer (pH 7.4). After centrifugation at 12,000 g for 20 min, the MPO activity in the supernatant was measured using the corresponding kits [13,17].

### Measurement of the GSH level

The collected tissue samples were homogenized in ice-cold 10% (w/v) trichloroacetic acid (TCA; 1 g of tissue in 10 mL of TCA) using a tissue homogenizer. Briefly, following centrifugation of the tissue at 3000 g for 10 min, 0.5 mL of supernatant was added to 2 mL of a 0.3 M Na_2_HPO_4_·2H_2_O solution. A 0.2-mL volume of a solution of dithiobis nitrobenzoate (0.4 mg in 1 mL of 1% sodium citrate) was added, and the absorbance was then measured at 412 nm.

### Statistical analysis

Significant differences between groups were determined by analysis of variance (ANOVA). Comparisons of the histological severity scores were made using the Kruskal–Wallis H test. A P value of less than 0.05 was considered to be statistically significant. The results are expressed as the means ± standard deviation (SD).

## Results

### H_2_ levels in the blood and media

The H_2_ levels in the arterial blood samples and media at the end of the *in vivo* and *in vitro* experiments were measured by gas chromatography. Inhalation of 2% H_2_ for 12 h resulted in a significant elevation in the arterial H_2_ level to 623±67.3 ng/mL. The medium in the H_2_ group had an average H_2_ level of 156.7±7.5 ng/mL. H_2_ could not be detected in the blood or media of the other groups. These results suggest that H_2_ may be systemically delivered to rodent tissues and AR42J cells via the methods described above.

### Effects of H_2_ on cytokine production in AR42J cells after cerulein treatment

**Fig 1:**
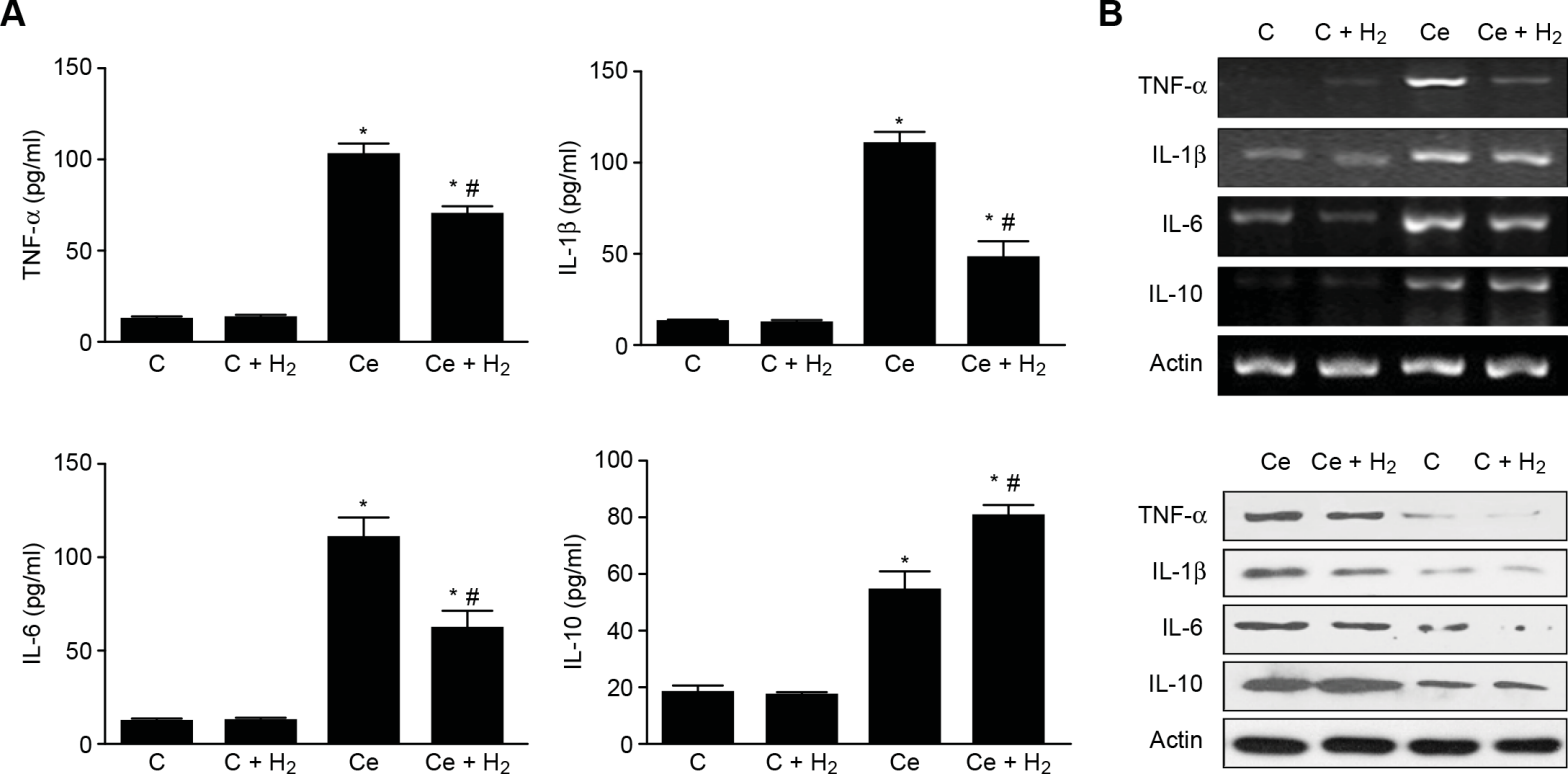
Effects of H_2_ on Cytokines Production in AR42J after Cerulein Treatment. (A) Cytokine levels in the culture medium. (B) Cytokine mRNA expression. (C) Cytokine protein levels. *P < 0.05 compared with the C group. #P < 0.05 compared with the Ce group.

The medium levels of TNF-α, IL-1β, and IL-6 were significantly increased in the Ce group (Fig 1A), and the H_2_ treatment significantly reduced these levels. The medium IL-10 level markedly increased after cerulein treatment and was significantly increased in the H_2_ group (Fig 1A). The expression of TNF-α, IL-1β, and IL-6 mRNA in AR42J cells was significantly increased after 24 h of cerulein treatment (Fig 1B). Compared with the sham group, in the Ce group, the TNF-α mRNA expression level was increased by 11-fold, the IL-1β mRNA expression level was increased by 22-fold, and the IL-6 mRNA expression level was increased by 302-fold. Treatment with H_2_ markedly decreased the expression of TNF-α, IL-1β, and IL-6 mRNA. A further analysis of anti-inflammatory cytokines showed that IL-10 mRNA expression was up-regulated by over 26-fold in the Ce group (Fig 1B), and treatment with H_2_ led to a further increase in the IL-10 expression level. A Western blot analysis confirmed that the expression levels of TNF-α, IL-1β, and IL-6 were significantly increased after induction with cerulein compared with the control groups. The administration of H_2_ significantly down regulated the expression of these three cytokines, whereas the expression of IL-10 showed the opposite trend (Fig 1C).

### Effects of H_2_ on pancreatitis indicators in cerulein-treated AR42J cells

**Fig 2:**
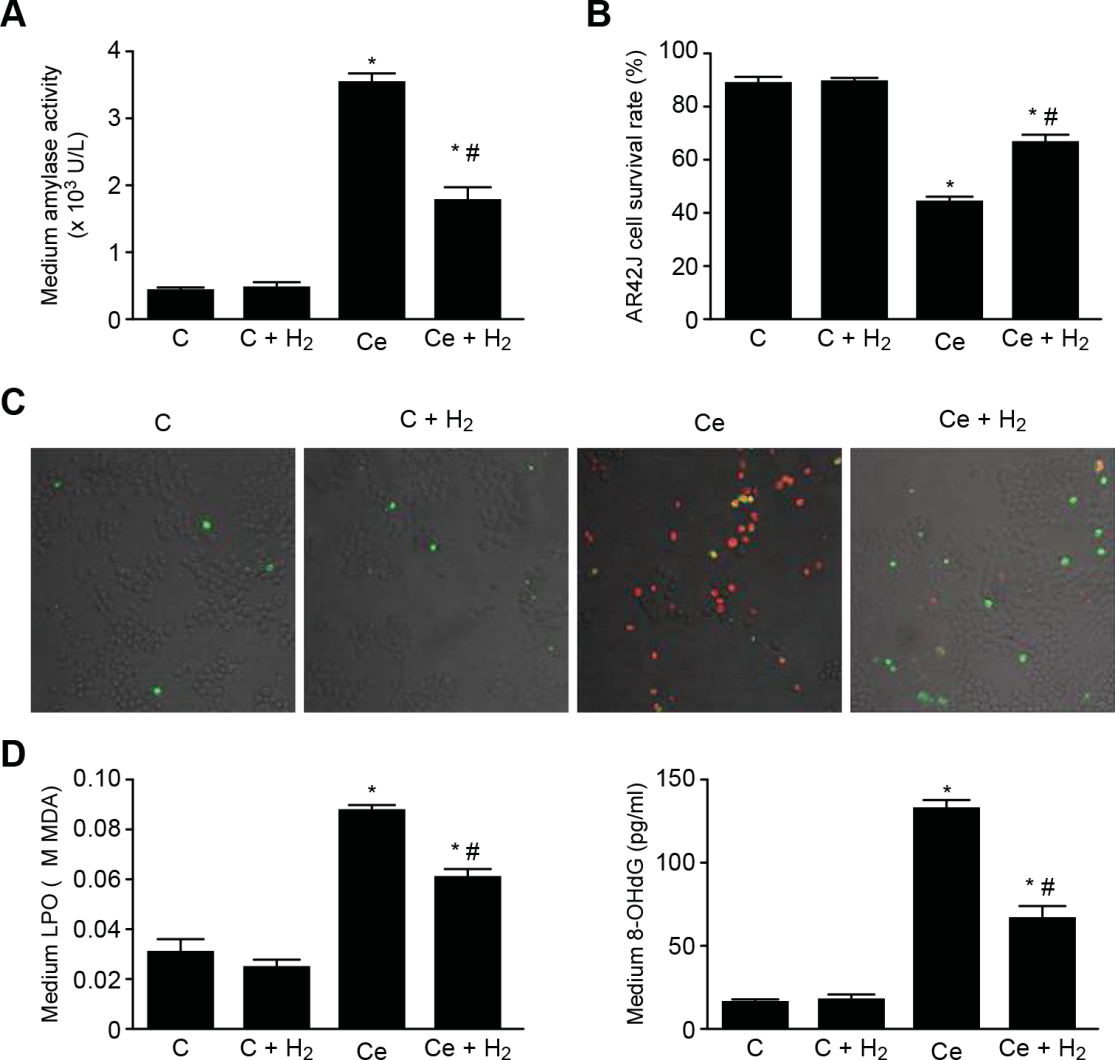
Effects of H_2_ on Pancreatitis Indicators in Cerulein-treated AR42J Cells. (A) Amylase activity. (B) Cell survival. (C) Cell apoptosis detected by Annexin V/PI staining (×200). (D) LPO production. (E) 8-OHdG level. *P < 0.05 compared with the C group. #P < 0.05 compared with the Ce group.

To examine the protective effect of H_2_ against cerulein in acinar cells, the amylase activity in the culture medium and cell survival were assessed. Compared with the C group, in the Ce group, the amylase activity was significantly higher, and cell survival was significantly lower. In addition, compared with the Ce group, the level of amylase activity was significantly decreased in the H_2_ group (Fig 2A), and cell survival was significantly higher in this group (Fig 2B). The effects of H_2_ on the cerulein-induced injury of AR42J cells were also assessed by laser scanning confocal microscopy (Fig 2C), which revealed that the number of necrotic cells was markedly increased in the Ce group and that treatment with H_2_ resulting in sparse necrotic cells. These results confirm that AR42J cells can be protected against cerulein-induced inflammatory damage by H_2_, which is consistent with the findings from the cytokine analysis. To examine the effect of H_2_ on cerulein-induced oxidative stress, the medium LPO and 8-OHdG levels were measured, and the results showed that the medium LPO and 8-OHdG levels were higher in the Ce group compared with the C group and significantly lower in the H_2_ group compared with the Ce group (Fig 2D and 2E).

### Effects of H_2_ on cytokine production in pancreatic tissues after AP induction

**Fig 3:**
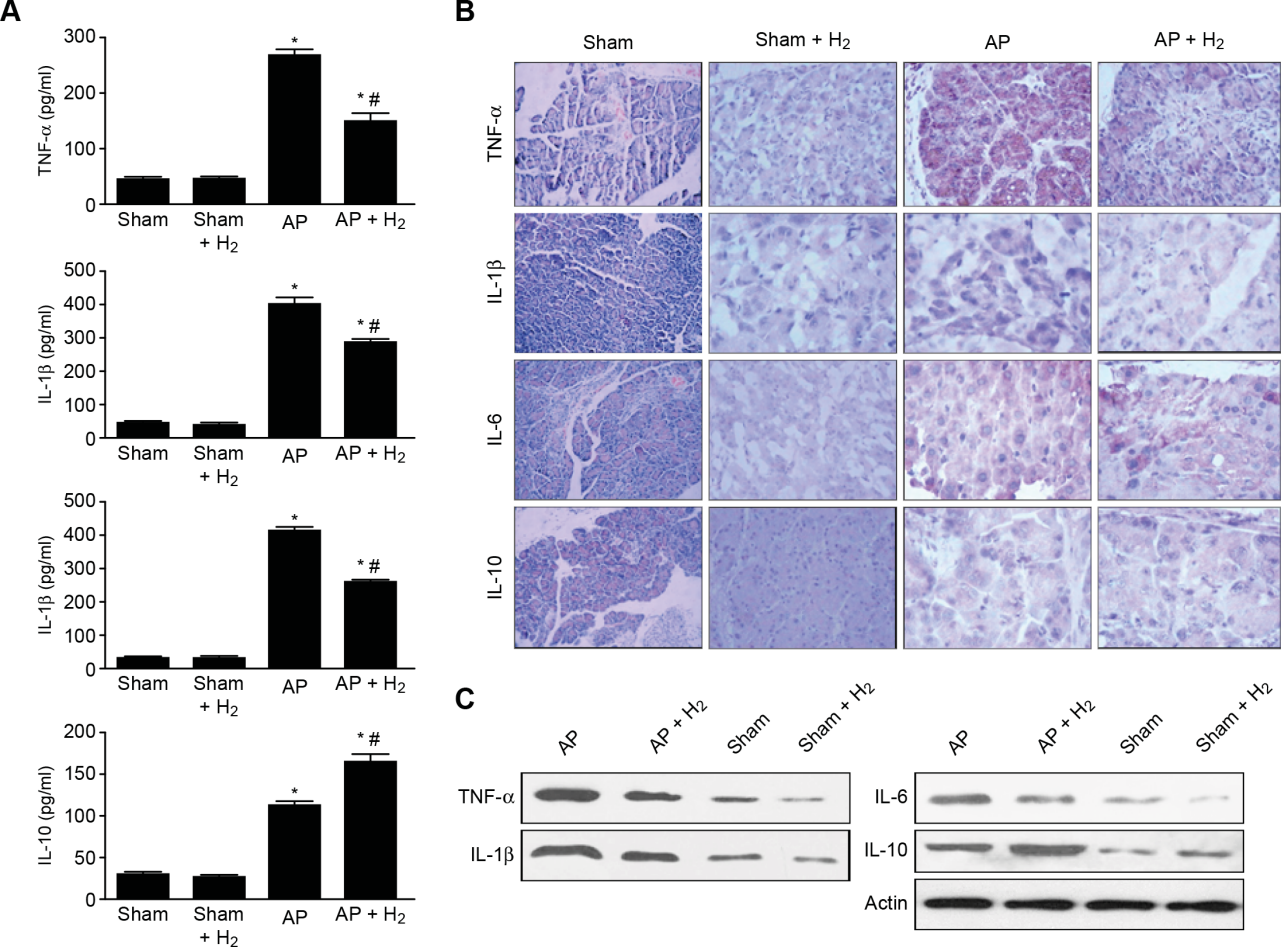
Effects of H_2_ on Cytokines Production in Pancreatic Tissues after AP Induction. (A) Cytokine levels in serum. (B) Immunohistochemistry detection of cytokine expression in pancreatic tissue (×400). (C) Cytokine protein levels in pancreatic tissue. *P < 0.05 compared with the sham group. #P < 0.05 compared with the AP group.

The serum levels of TNF-α, IL-1β, and IL-6 were significantly increased 12 h after the onset of AP (Fig 3A), and the H_2_ treatment significantly reduced these levels. In contrast, the serum IL-10 level was markedly increased after the induction of AP and was significantly increased in the H_2_ group (Fig 3A). The trendency in the variations in the levels of the cytokines listed above was verified by immunohistochemical analysis. The protein levels of TNF-α, IL-1β, and IL-6 in pancreatic tissue were significantly increased 12 h after the onset of AP (Fig 3B), and the H_2_ treatment significantly reduced the protein levels of TNF-α, IL-1β, and IL-6. The IL-10 level significantly increased after the induction of AP and was also significantly increased in the H_2_ group (Fig 3B). A Western blot analysis demonstrated that the expression levels of TNF-α, IL-1β, and IL-6 were markedly elevated after induction with taurocholate compared with the control. The administration of H_2_ significantly decreased the expression levels of these cytokines, whereas IL-10 exhibited the opposite trend (Fig 3C).

### Effects of H_2_ on pancreatic function in rats with taurocholate-induced AP

**Fig 4:**
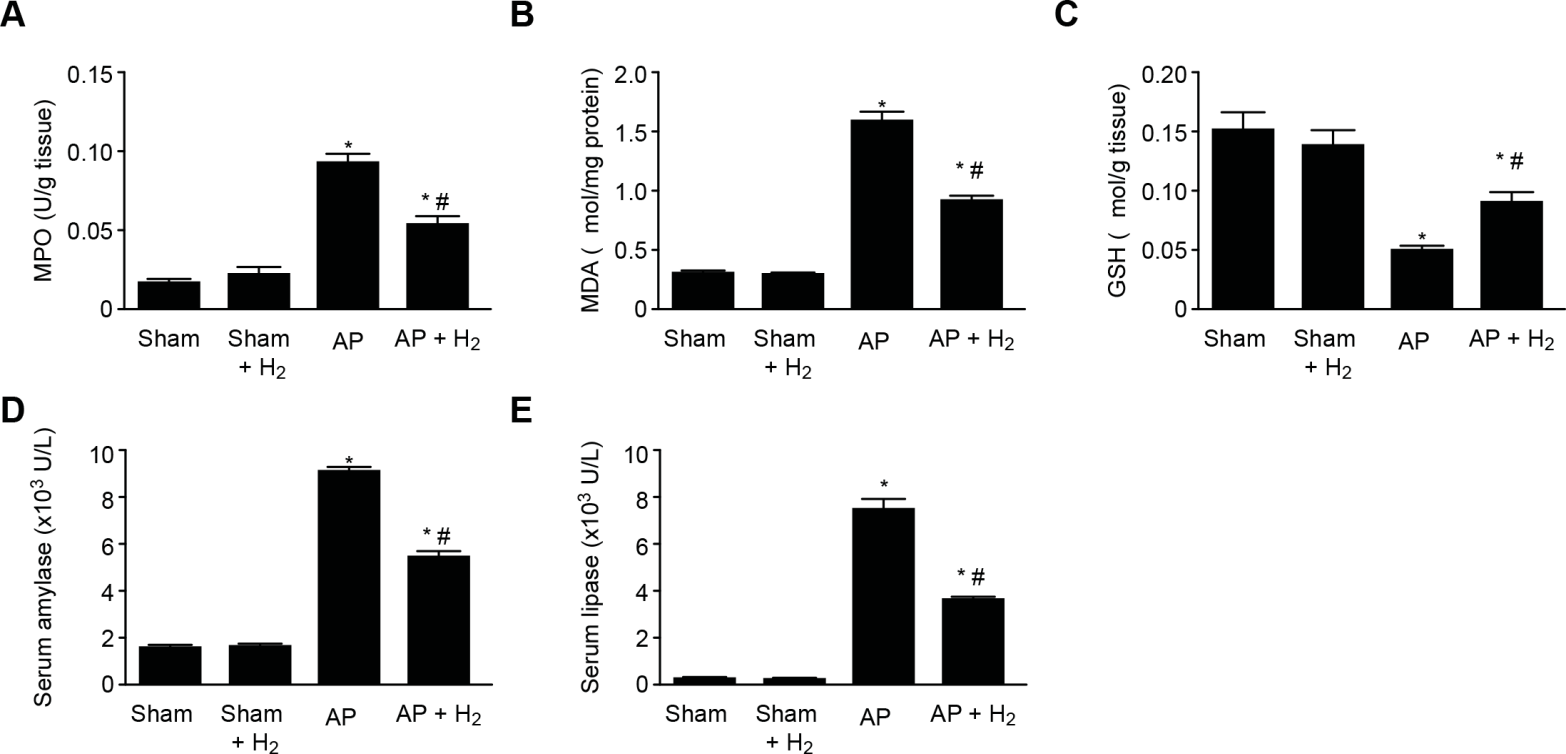
Effects of H_2_ on Pancreatic Function in Taurocholate-induced AP Rats. (A) MPO level. (B) MDA production. (C) GSH level. (D) Serum amylase level. *P < 0.05 compared with the sham group. #P < 0.05 compared with the AP group.

AP was induced in rats through a retrograde intraductal infusion of 5% sodium taurocholate. Measurement of MPO activity revealed that the increase induced by taurocholate infusion was significantly attenuated by treatment with H_2_ (Fig 4A). To confirm the involvement of oxidative stress in the H_2_-associated protection against taurocholate-induced AP, the MDA and GSH levels were assessed. The pancreatic MDA level was significantly higher in the AP group than in the sham group but was significantly lower in the H_2_ group (Fig 4B). In addition, the pancreatic GSH level was lower in the AP group compared with the sham group but was significantly higher in the H_2_ group compared with the AP group (Fig 4C). The serum amylase and lipase levels were significantly increased in the rats of the AP group following the onset of AP (Fig 4D and 4E), and the inhalation of H_2_ significantly decreased the serum amylase and lipase levels in these rats (Fig 4D and 4E).

### Effects of H_2_ on pancreatitis in rats with taurocholate-induced AP

**Fig 5:**
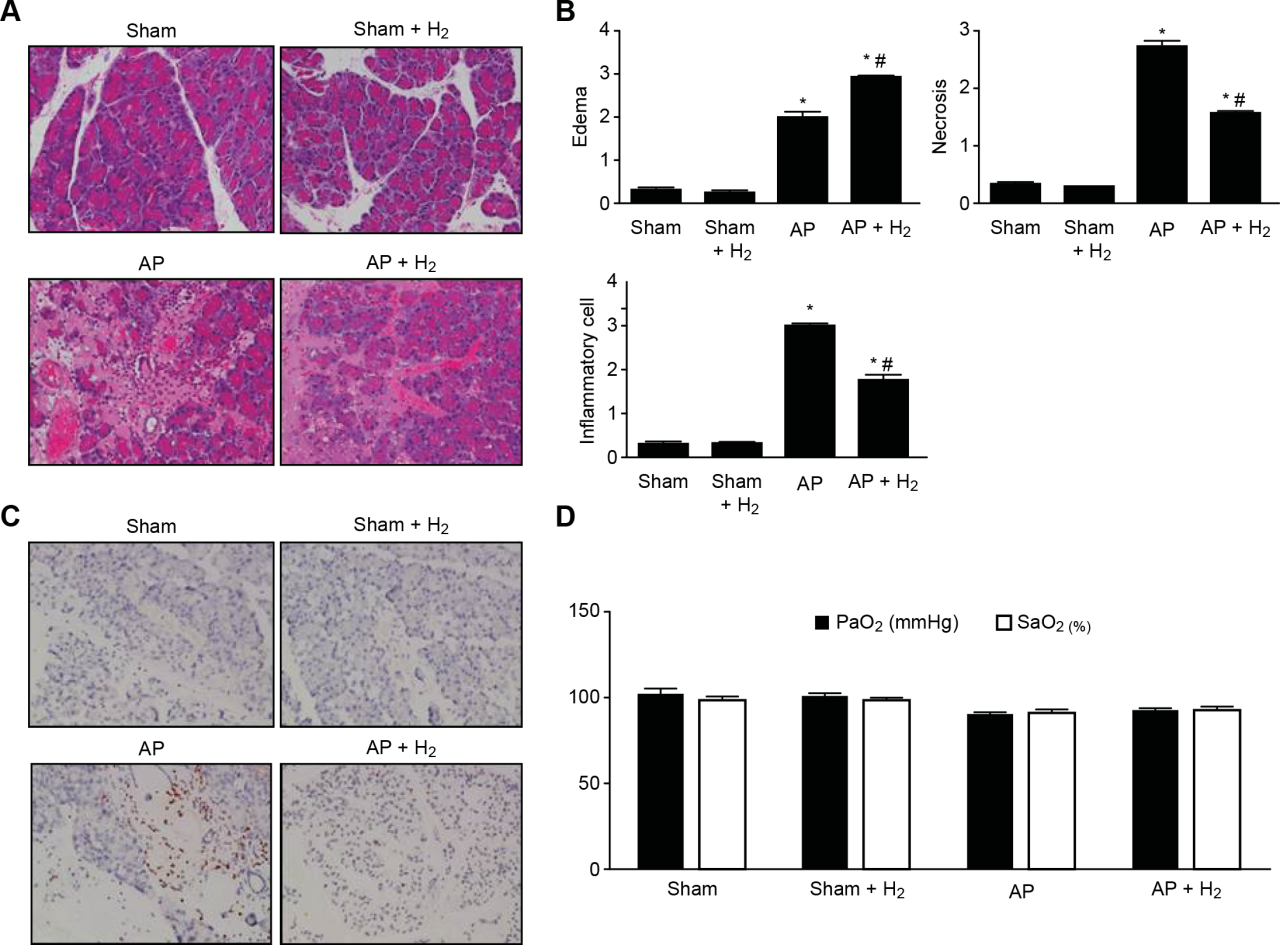
Effects of H_2_ on Pancreatitis in Rats with Taurocholate-induced AP. (A) H&E staining of pancreatic injury (×400). (B) Histological score. (C) Neutrophil infiltration (×400). (D) Blood gas analysis. *P < 0.05 compared with the sham group. #P < 0.05 compared with the AP group.

The severity of pancreatitis was also examined by histological analysis. The pancreases from the rats in the sham group exhibited a normal architecture (Fig 5A), whereas taurocholate-induced pancreatic damage was obvious in the rats in the AP group following taurocholate injection (Fig 5A), and this pancreatic damage was decreased in the pancreases of the rats in the H_2_ group (Fig 5A). The pathological scoring of pancreatic damage revealed that the taurocholate treatment resulted in significant increases in edema, inflammatory cell infiltration, and necrosis compared with the sham group (Fig 5B) and that the treatment of the pancreatitic rats with H_2_ markedly reduced the severity of AP (Fig 5B). Because neutrophil infiltration is an important evidence of pancreatitis, we assessed the neutrophils in rat pancreatic tissue by immunohistochemistry, which demonstrated that H_2_ significantly alleviated AP-induced neutrophil infiltration without affecting the number and function of neutrophils observed under normal conditions (Fig 5C). In addition, even though AP treatment slightly decreased the PaO_2_ and SaO_2_ levels, H_2_ treatment did not have a marked impact on these levels (Fig 5D).

## Discussion

The early stage of SAP is recognized as the first death fastigium. Patients should be provided meticulous treatment in an intensive care unit during this period. However, satisfactory therapeutic results have not been achieved in these patients. The pathophysiological mechanisms responsible for SIRS seem to differ from those involved in local pancreatic damage. The crosstalk between cytokines and oxidative stress is responsible for the conversion of a local inflammatory process into a systemic inflammatory response and appears to be the major factor responsible for the development of SIRS [22]. Therefore, treatments that inhibit cytokine production or neutralize cytokines with antioxidants have been proven to have slight beneficial effects in experimental AP.

H_2_ is universally acknowledged as a potent free radical scavenger. Many studies have shown the protective effect of H_2_ on tissue damage, including intestinal graft injury, myocardial injury and renal injury induced by oxidative stress [23–25]. In addition, a series of experiments have demonstrated that H_2_ has potential anti-inflammatory effects because it can reduce the up-regulation of cytokines [26–28].

H_2_ has unique properties compared with other free radical scavengers. (1) Because it is the smallest molecule in nature, it is permeable to cell membranes and can target organelles, including the mitochondrion and nucleus. (2) H_2_ specifically quenches detrimental reactive oxygen species (ROS), such as ·OH and ONOO^-^, while maintaining metabolic oxidation–reduction reactions and levels of other less potent ROS, such as O_2_^-·^ and H_2_O_2_. (3) The end product of the reaction of H_2_ with ROS is water, which is nontoxic and harmless [10].

Previous studies mainly focused on animal models. However, the animals were treated with hydrogen-water, leading to a low hydrogen concentration in the body and rapid decreases in the blood hydrogen concentration. In our study, hydrogen was administered via inhalation, and the blood hydrogen concentration was monitored using a gas chromatograph. This approach can maintain the hydrogen concentration at a sustained high level to guarantee that hydrogen exerts an effect in the body. Furthermore, a gas chromatograph was also used to monitor the hydrogen concentration in the medium in the *in vitro* experiment performed in this study, which thoroughly tested the effect of hydrogen on pancreatic cells.

Based on the unparalleled advantages of H_2_, this research was designed to assess its effects on cytokines and oxidative stress in AP both *in vivo* and *in vitro*. To the best of our knowledge, the effects of H_2_ gas on AP in rats and AR42J cells have not been comprehensively and simultaneously assessed in a previous study. In this study, we found that taurocholate infusion and cerulein treatment result in highly elevated serum amylase and lipase levels after the induction of AP. The treatment of the same subjects with H_2_ yielded marked reductions in these levels, and the medium amylase activity also appeared to be significantly decreased.

Tissue MPO activity is frequently evaluated to estimate polymorphonuclear neutrophil (PMN) accumulation in inflamed tissue, and MPO activity is significantly correlated with the number of histologically determined PMNs in tissue. In this study, we found that the pancreatic MPO activity was markedly elevated after AP induction and significantly attenuated by the administration of H_2_. This finding indicated that H_2_ effectively prevented PMN chemotaxis and infiltration in the pancreas after AP and consequently decreased pancreatic injury. This conclusion was also verified by histological evaluation and immunohistochemical analysis.

The process of pancreatic damage induces a local inflammatory reaction that results in SIRS and distant organ failure. This damage is mediated by different cytokines and free radicals released during the course of SAP. TNF-α and IL-1β are pivotal cytokines in AP that exhibit synergic effects in the amplification of the inflammatory response. IL-6 is the primary inducer of the acute-phase protein response [29]. The serum level of IL-6 is considered a marker for the severity of AP, although it does not form the basis for the initiation and propagation of the systemic inflammatory response. This study revealed that the induction of AP was accompanied by increases in the release of TNF-α, IL-1β, and IL-6 into the serum and medium. Significant increases in the mRNA expression levels of these cytokines were also evident in the AR42J cells. We found that treatment with H_2_ significantly suppressed the gene expression of these cytokines and decreased their serum and medium levels. Different from these pro-inflammatory cytokines, IL-10 is recognized as a potent anti-inflammatory cytokine with immunosuppressive properties [17]. Significant increases in IL-10 levels in both the serum and medium were observed in this study, and the same finding was obtained from the analysis of IL-10 mRNA expression after the development of AP. The administration of H_2_ not only significantly increased the serum and medium IL-10 levels but also increased the IL-10 mRNA level. These findings support the hypothesis that H_2_ exerts potent anti-inflammatory effects on AP. The above-mentioned conclusions were confirmed by immunohistochemistry and Western blot analyses.

Oxidative stress is defined as a disturbance in the balance between pro-oxidants and antioxidants and appears to play a significant role in AP because it is related to the severity of this condition. The marked increase in free radicals is correlated with the tissue injury that occurs in AP and may be confirmed by an increase in the MDA level [30]. The lipid peroxide levels also increase in the blood of animals with AP, and these changes are associated with the severity of the disease [31,32]. In this study, the LPO levels were noticeably increased both *in vivo* and *in vitro* after the induction of AP, but this activity was significantly decreased in the H_2_ group. GSH plays a central role as an antioxidant in AP. GSH depletion in pancreatic tissue is a hallmark of oxidative stress during the early phase of AP [33] and may contribute to the progression from mild to severe AP [6]. In the current study, the pancreatic GSH levels were low in the AP rats but appreciably increased after H_2_ treatment. Oxidative stress is one of the major causes of DNA damage and death in acinar cells. The oxidative DNA damage marker 8-OHdG was measured in cerulein-treated AR42J cells, and its level in the medium was found to be significantly reduced after treatment with H_2_. In addition, the AR42J cell survival rate was assessed to verify the protective effect of H_2_ against AP, and the results show that cell survival was markedly higher following treatment with H_2_. These findings were supported by results obtained through laser-scanning confocal microscopy, which revealed a marked increase in the number of necrotic cells after the induction of AP, whereas treatment with H_2_ resulted in only sparse necrotic cells. The protective effect of H_2_ against oxidative DNA damage may be responsible for the down-regulation of cytokines.

Taken together, our results indicate that a complex and dynamic inflammatory network forms after the induction of AP and gives rise to different interactions between cytokines and oxidative stress that depend on the type of cell. The therapeutic treatment of AP with H_2_ might confer excellent advantages via its effects on cytokines and oxidative stress. The findings revealed that H_2_ can help protect against acute pancreatitis attack.

In conclusion, this study discovered that H_2_ gas significantly reduces the inflammatory cytokine levels and oxidative damage caused by AP both *in vivo* and *in vitro* and improves the antioxidative abilities of pancreatic tissue, thereby conferring protection against AP. H_2_ gas has a potentially wide range of applicability for AP treatment. Consequently, further studies are necessary to reveal the detailed mechanisms underlying the protective effects of molecular H_2_ against AP, which may reveal new potential therapeutic approaches for this severe disease.
